# Properties of Immature Myeloid Progenitors with Nitric-Oxide-Dependent Immunosuppressive Activity Isolated from Bone Marrow of Tumor-Free Mice

**DOI:** 10.1371/journal.pone.0064837

**Published:** 2013-07-02

**Authors:** Parvin Forghani, Wayne Harris, Cynthia R. Giver, Abbas Mirshafiey, Jacques Galipeau, Edmund K. Waller

**Affiliations:** 1 Department of Pathobiology, School of Public Health, Tehran University of Medical Sciences, Tehran, Iran; 2 Department of Hematology and Medical Oncology, Winship Cancer Institute, Emory University, Atlanta, Georgia, United States of America; University Hospital of Heidelberg, Germany

## Abstract

Myeloid derived suppressor cells (MDSCs) from tumor-bearing mice are important negative regulators of anti-cancer immune responses, but the role for immature myeloid cells (IMCs) in non-tumor-bearing mice in the regulation of immune responses are poorly described. We studied the immune-suppressive activity of IMCs from the bone marrow (BM) of C57Bl/6 mice and the mechanism(s) by which they inhibit T–cell activation and proliferation. IMCs, isolated from BM by high-speed FACS, inhibited mitogen-induced proliferation of CD4^+^ and CD8^+^ T-cells *in vitro*. Cell-to-cell contact of T-cells with viable IMCs was required for suppression. Neither neutralizing antibodies to TGFβ1, nor genetic disruption of indolamine 2,3-dioxygenase, abrogated IMC-mediated suppressive activity. In contrast, suppression of T-cell proliferation was absent in cultures containing IMCs from interferon-γ (IFN-γ) receptor KO mice or T-cells from IFN-γ KO mice (on the C57Bl/6 background). The addition of NO inhibitors to co-cultures of T-cells and IMC significantly reduced the suppressive activity of IMCs. IFN-γ signaling between T-cells and IMCs induced paracrine Nitric Oxide (NO) release in culture, and the degree of inhibition of T-cell proliferation was proportional to NO levels. The suppressive activity of IMCs from the bone marrow of tumor-free mice was comparable with MDSCs from BALB/c bearing mice 4T1 mammary tumors. These results indicate that IMCs have a role in regulating T-cell activation and proliferation in the BM microenvironment.

## Introduction

Normal BM contains a heterogeneous population of cells, including immature macrophages, granulocytes, and myeloid dendritic cells. Immature myeloid cells, termed myeloid-derived suppressor cells (MDSC), are expanded in tumor-bearing mice, and in patients with cancer, trauma, and autoimmunity [1], [2]. The phenotype of MDSCs described in different tumor models is based upon their expression of one or more myeloid markers including the β2 integrin CD11b [3], MCSF-R [4], CD124 [5], and the two epitopes of the myeloid marker GR-1, Ly6C [6] and Ly6G [7]. While an immunosuppressive role of tumor-associated MDSCs has been well characterized in murine models [8], the immuno-modulatory functions of BM-derived CD11b^+^GR-1^+^ IMCs from normal mice has not been well studied. Ly6G^+^ Ly6C^low^ granulocytic and Ly6G^+^Ly6C^hi^ monocytic MDSCs, both of which inhibit T-cell activation and proliferation in tumor-bearing mice via NO-dependent and NO-independent mechanisms [9], [10]. Although a number of reports have described natural suppressor (NS) myeloid cells in BM from non-tumor bearing animals [11], [12], most studies during the last 20 years have focused on tumor-associated myeloid-derived suppressor cells and suggest that BM-derived CD11b^+^ GR-1^+^ immature myeloid cells (IMCs) in normal, tumor-free mice lack immunosuppressive activity [8], [13]. Thus, the role of IMCs in the immune homeostasis of non-tumor bearing animals remains only partially understood. The BM is a common site for tumor metastasis for a variety of cancers, suggesting that the BM environment maybe immuno-suppressive and limit adaptive immune responses to metastatic cancer. The current study was undertaken to characterize the immuno-suppressive activity of myeloid precursors from the BM of non-tumor-bearing mice. To do this, we isolated different populations of immature myeloid cells and studied their immune suppressive effect on T ell proliferation *in vitro*. We report herein that CD11b^+^GR-1^+^ lineage (Lin)^−^ cells from normal BM have potent suppressive activity *in vitro*, limiting Dynabeads induced T-cell proliferation. The IMC population consists of GR-1^hi^ and GR-1^int/low^ subsets of myeloid cells with suppressive activity comparable to MDSCs isolated from 4T1 tumor-bearing mice. Functional analysis of IMCs from the BM showed that IFN-γ and (NO) signals are critical in the suppression mediated by IMCs.

## Materials and Methods

### Mice and cell lines

BALB/c mice, C57Bl/6, BA (CD90.1 congenic strain of B6), and genetic knockout strains (KO) on the C57Bl/6 background (IDO KO, IFN-γ KO, IFN-γ receptor KO and IL-12 KO) were purchased from the Jackson Laboratory (Bar Harbor, ME). Mice were housed under specific-pathogen free conditions and used between 2 and 6 months of age. Animal experiments were carried out under an Institutional Animal Care and Use Committee (IACUC). The protocol was approved by the committee on the Ethics of animal Experiments of the university of Emory (Permit number: A3180-01). LBRM 33-5A4, a B10-derived T-cell lymphoblastic lymphoma cell line [14] and the 4T1 breast cancer cell line, derived from BALB/c were purchased from ATCC. Cells were cultured in RPMI supplemented with 10% fetal bovine serum, 0.4 mmol/L sodium pyruvate and antibiotics (penicillin, streptomycin). 4T1 cells (1×10^6^ in 100 µL PBS) were injected subcutaneously into the right flank of female BALB/c mice.

### Preparation of bone marrow cells and splenocytes

Bone marrow cells (BMC) were harvested from the femur bones of mice, cutting off each end, and flushing out the bone marrow with PBS. Splenocytes and BMC were passed through a 70 µm nylon mesh cell strainer [15], pelleted, re-suspended in red blood cell lysis buffer, followed by washing in PBS. T-cells were purified by negative selection after incubating splenocytes with biotinylated anti-CD11b, B220 (B cells), DX5 (NK cells), and Ter119 (RBCs) antibodies using an LS immunomagnetic column system (MACS, Miltenyi Biotech) according to the manufacturer's instructions. T-cell purity was 85–90% as determined by FACS.

### Antibodies and Reagents

Monoclonal antibody (Mab) details are provided in Table 1. Neutralizing antibodies against TGF-β1 (clone 2AR2, 1.5–5 µg/ml), and IL-4 (clone 11B11, 50 µg/ml) IL10 (clone JES5-16E3, 5 µg/ml), were purchased from eBioscience (Table 1). INOS inhibitors L-NG-monomethyl arginine (L-NMMA, Sigma-Aldrich, St. Louis), L-NIL, (N6- (1-iminoethyl)-lysine, hydrochloride, Biotium, Inc, USA) and L-NIO (N5- (1-iminoethyl)-L-ornithine, dihydrochloride, Biotium) were added to T-cell-IMC cultures.

**Table 1:** Conjugated monoclonal antibodies.

PE	Clone	Used Form	Company
CD11b/Mac-1	M1/70	APC-Cy/Biotin	BD PharMingen
CD11c	HL3	APC	BD PharMingen
CD115	AF-596	PE	e Bioscience
F4/80	BM8	PE	e Bioscience
CD86	Gl1	PE	e Bioscience
Ly6C	AL-21	PE	BD Pharmingen
Nk1.1/DX5	Pk136	PE/Biotin	Pharmingen
Ly6G	1AB	PE	BD Pharmingen
PDL2	Ty25	PE	e Bioscience
CD45R (B220)	RA3-6B2	PE/Biotin	e Bioscience
CD3	145-2C11	APC	BD PharMingen
CD80	16-10A1	PE	e Bioscience
I-A [b]	AF6-120.1	PE	BD PharMingen
H2 kb	AF6.88.5	PE	BD PharMingen
CD25	Pc61	APC-Cy	BD bioscience
CD8	53-6.7	APC-Cy/PE	BD bioscience
GR-1	RB6-8c5	PE/FITC	BD PharMingen
PDL-1	M1H5	PE	BD Pharmingen
TER-119	TER-119	PE/Biotin	BD Pharmingen
Ki-67		PE	BD Pharmingen
Isotype IgG1 K		PE	BD Pharmingen
Isotype IgG2a K		PE	BD Pharmingen
Isotype IgG2b		PE	BD Pharmingen
Purified Rat anti mouse IL-10			BD Pharmingen
Purified Rat anti mouse IL-4			Abcam
Purified Rat anti mouse to TGF β			BD pharmingen

### Cell sorting

BM cells were washed with 1× PBS, FC blocked with anti-CD16/CD32 Mab (Fc Block, BD Bioscience) prior to incubation with 2 µg/ml anti-CD11b (APC-Cy7) and anti-GR-1 (FITC) Mabs in combination with a pool of PE-conjugated lineage Mabs against Ter-119, CD3, CD45R and DX-5 in 1× PBS with 1% BSA for 20 min at 4°C. After washing with 1× PBS, cells were resuspended in RPMI-1640 supplemented with 10% FBS and 1% penicillin/streptomycin, and sorted using a FACS Aria cell sorter and FACSDiva software version 5.2 (BD Biosciences, San Jose, CA). Flow cytometric reanalysis of the sorted cells showed 92.4%±0.5% and 96.4%±3% purity for CD11b^+^GR-1^hi^ and CD11b^+^GR-1^int/lo^ IMCs, respectively.

### Morphological Analysis

Cytospin slide preparations of sorted purified cells were made using a Shandon Southern Cytospin (Thermo Scientific, Walthman, MA). Giemsa staining (1/20 dilution) was done for 30 minutes and cells were photographed at 63× using a Zeiss axioplan 2 upright microscope (Carl Zeiss, Inc).

### Carboxyfluorescein diacetate succinimidyl diester (CFSE) labeling and Proliferation Assay

Staining conditions were optimized to distinguish proliferated cells from undivided cells. CFSE (Invitrogen Corporation, Carlsbad, CA) was diluted in DMSO at a stock concentration of 5 mM, which was then diluted to the final working concentration of 1 µM. Whole splenocytes or T-cells (1×10^6^ cells/ml) were stained using 1 µM CFSE in PBS for 10-min at 37°C. Staining was quenched by the addition of two volumes of ice-cold RPMI-1640 (10% FBS, 1% penicillin/streptomycin) and incubation on ice for 5 min in RPMI and 10% FBS. Stained cells were washed twice and resuspended to the desired concentration. Freshly CFSE labeled T-cells were co-cultured at a density of 1×10^6^/per well with or without fresh CD11b^+^GR-1^+^ cells (ratio 1/1) in the presence of anti-CD3/CD28 Dynabeads, or with CON A (250 ng/ml). In some experiments, sorted IMCs were fixed in 1% Para formaldehyde (PFA), pH 7.0 for 1 hour at 4°C and washed several times prior to co-culture with T-cells. Cell–cell contact dependency was evaluated using a 0.4 µm pore size Transwell plate (Corning Costar, Cambridge, MA). After 5 days, cultured T-cells were harvested, stained with fluorochrome-conjugated anti-CD3, anti-CD4 and anti-CD8 antibodies, followed by the addition of 7-amino-actinomycinD (7-AAD), then analyzed by flow cytometry to determine the CFSE profiles of viable (7-AAD negative) T-cell populations, acquiring at least 150,000. The proliferation index (PI) of CD4 or CD8 T-cells was determined by analyzing the CFSE histograms [16]. Inhibition of proliferation was calculated as follows: [100−(PI sample/PI control)*100 = % suppression]. The percentages of apoptotic and dead cells were quantified using Annexin V and 7-AAD (BD Pharmingen).

### Extracellular Nitric oxide measurement

Culture supernatants were mixed with Griess reagent (Molecular Probes, Eugene, OR) based on the manufacturer's instructions and absorbance was measured at 570 nm using a microplate plate reader (Epoch-BioTek). Nitrite concentrations were determined by comparing the sample absorbance values versus a standard curve generated by serial dilution of 50 mM sodium nitrite.

### Intra cellular assay of Nitric oxide

To measure intracellular NO, DAF-FM (4-amino-5-methylamino- 2,7-difluorescein) (Molecular Probes, OR, USA) was used. DAF-FM readily diffuses into cells and within the cytoplasm releases DAF-FM by the action of intracellular esterase. DAF-FM is essentially non-fluorescent until it is nitrosylated by products of oxidation of NO, resulting in DAF-FM triazole which exhibits about a 160- fold greater fluorescence quantum efficiency at 495 nM. Un-labeled activated T-cells were cultured in presence and absence of naïve BM-derived sorted CD11b^+^ cells. After 4 days cells were harvested from culture, washed with PBS, incubated with 10 µM DAF-FM for 45 min at 37°C, washed once with PBS and then incubated for additional 20 minutes to complete de-esterification of intracellular di-acetates.

### Data analysis

Statistical analyses were performed using Graph Pad Prism Software (OS X version 5.00 for Macintosh, Graph Pad, San Diego, California, USA). Differences between groups were analyzed using unpaired two-tailed Student's *t* test and Mann-Whitney test. *P*<0.05 was considered statistically significant. Quantitative data are presented as mean ± SD.

## Results

### BM of non-tumor-bearing mice contains CD11b^+^GR-1^+^ IMCs with potent suppressive activity that limits T-cell activation and proliferation

**Figure 1:**
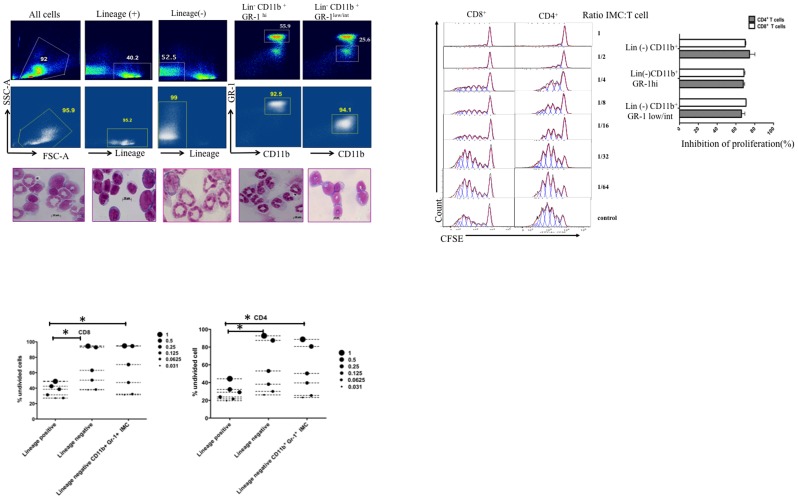
Suppressor activity of bone marrow myeloid cell subsets. Single cell suspensions of bone marrow were prepared from C57BL/6 mice and stained with anti-CD11b APC-Cy, anti GR-1 FITC, and lineage PE cocktail. Lineage (+), lineage (−), CD11b^+^GR-1^hi^ and CD11b^+^GR-1^low/int^ BM populations were isolated by FACS. A) Pre sort gates (upper panels). Reanalysis of sorted populations (middle panels). Lower panels represent morphology of Giemsa stained cells (63× magnification). B) CFSE fluorescence histograms of viable 7-AAD (−) MACs purified T-cells co-cultured with different CD11b^+^ GR-1^+^/splenocyte ratios (left panel). C) Comparison of the percentage inhibition of proliferation of T-cells co-cultured with CD11b^+^GR-1^hi^, CD11b^hi^GR-1^low/int^ and lineage (−) CD11b^+^ cells (ratio1/1). Bars represent the mean values ± SD of two experiments. D) Comparison of the potency of sorted BM fractions of IMCs (mix of both CD11b^+^GR-^1hi^, CD11b^hi^GR-1^low/int^) on percentage of undivided CFSE labeled T-cells following 5 days co culture in the presence of anti-CD3/CD28 beads, and IL-2. The legend shows the ratio of sorted IMC: T-cells with the size of the symbol representing the relative numbers of IMCs in the culture. *P* value<0.05 represent significant difference for both percentage of undivided CD4^+^ and CD8^+^ T-cells between lineage positive with lineage negative and CD11b^+^ GR-1^+^ IMCs at (IMC: T ratios of 1 and 0.5). Data from a single experiment, representative of 4 individual experiments, is shown.

**Figure 2:**
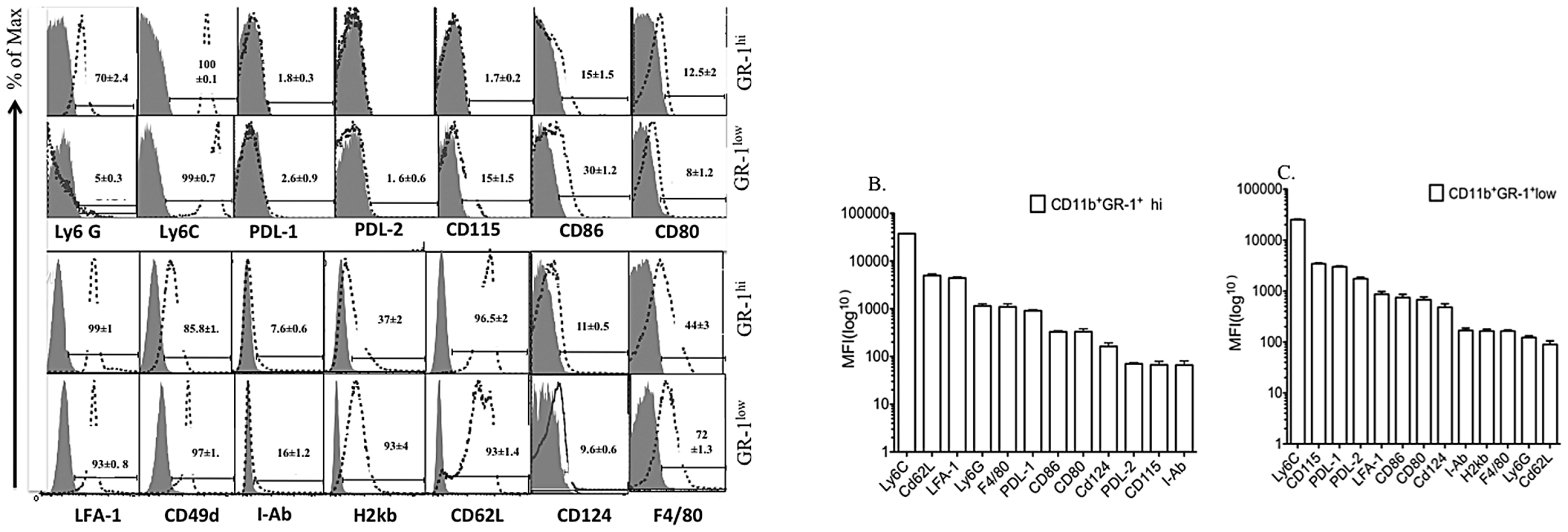
Expression of surface molecules on BM-derived CD11b^+^GR-1^+^ IMC subsets. Flow cytometry analysis of cell surface marker expression on lineage (−) CD11b^+^GR-1^hi^ and CD11b^+^GR-1^low/int^ IMC subsets was performed as described in the Methods section. Histograms represent expression of the indicated markers on viable CD11b^+^GR-1^+^ cells (open dashed histograms) compared with gated isotype control (filed gray histograms). Data represent of average of frequencies (± SD) from replicate samples. B) Logarithmic mean fluorescence index (MFI) of three experiments for both subsets of CD11b^+^GR-1^hi^ and CD11b^+^GR^−/low/int^ IMCs respectively (B & C) ordered by marker from the greatest to the least mean MFI.

To assess the potential of BM-derived CD11b^+^GR-1^+^ IMCs to limit T-cell activation and proliferation, we purified IMCs from BM of tumor-free mice that had a phenotype comparable to that of MDSC from tumor-bearing mice. Single cell suspensions of BM were prepared from naive C57BL/6 mice and stained with a cocktail of lineage markers, CD11b and GR-1. We used fluorescence-activated cell sorting (FACs) to isolate the following BM populations: all nucleated cells (gated by scatter parameter), lineage (+) cells, lineage (−) cells, lineage (−) CD11b^+^ GR-1^+^ IMC, and lineage (−) GR-1^hi^ and GR-1^low/int^ subpopulations of IMCs. 45–65% of lineage (−) BM cells were CD11b^+^GR-1^hi^ whereas 15–25% expressed CD11b with intermediate/low level of GR-1. Morphological analysis showed that CD11b^+^GR-1^hi^ lineage negative BM cells are mostly Ly6G^+^ immature granulocytic precursors while the CD11b^+^GR-1^low/int^ cells were CD115^+^ Ly6G^−^ monocytic precursors (Fig. 1A). To characterize the immunosuppressive properties of myelo-monocyte progenitors from non tumor- bearing mice, we isolated the GR-1^+^ granulocytic and GR-1^+^ monocytic subpopulations of IMCs by. We cultured the total population of lineage (−) cells, and two subpopulations of lineage (−) CD11b^+^GR-1^int^/^low^ monocytic cells and CD11b^+^GR-1^hi^ granulocytic IMCs with CFSE labeled splenocytes in the presence of anti-CD3/CD28 Dynabeads, and measured T-cell proliferation 5 days later. To quantitate the suppressive potency of subpopulations of IMCs from BM, we added graded numbers of FACS-sorted monocytic and granulocytic cells to a fixed number of T-cells (Fig. 1B). The lineage (−) CD11b^+^GR-1^+^ IMCs, comprising 25–35% of lineage negative BM, had greater T-cell suppressive activity compared with lineage positive BM cells. Both lineage (−) CD11b^+^ GR-l^hi^ and lineage (−) CD11b^+^GR-l^int^ IMC populations had equivalent capacity to suppress the proliferation of T-cells induced by anti-CD3/CD28 Dynabeads (Fig. 1C) and ConA activated T-cells (data not shown). FACS-purified lineage (−) BMCs and CD11b^+^ GR-1^+^ IMCs had more suppressive activity than lineage (+) BMCs (Fig. 1D). In order to evaluate the effect of IMCs on T-cell activation as well as T-cell division, we analyzed CD25 and Ki-67 expression on T-cells cultured alone and with CD11b^+^GR-1^+^IMCs. Our data showed higher expression of CD25 on T-cells after activation with anti-CD3/CD28 Dynabeads compared with T-cells co-cultured with IMCs (Fig. S1A). To understand the effect of IMCs on the viability of T-cells, cultures were stained with Trypan blue. We found a modest decrease in viable cells containing both T-cells and IMCs in contrast to expansion of viable cells in wells containing bead-activated T-cells alone (Fig. S1 B & S1 C). CFSE divided T-cells from control bead-activated cultures had more than 5-fold higher Ki-67 expression compared with CFSE divided T-cells that had been co-cultured with CD11b^+^ GR-1^+^ IMC sorted cells, indicating that IMC suppress both T-cell activation and cell division (Fig. S2). Immunophenotype analysis of BM-derived CD11b^+^GR-1^+^IMCs showed co-inhibitory markers (PDL-1& PDL-2), MHC class II, and co-stimulatory molecules CD80, CD86 were absent on both mononcytic and granulocytic IMC subpopulations (Fig. 1A). Expression of CD49d was higher on the CD11b^+^GR-1^int^ monocytic fraction, and expression of LFA-1 and CD62L were equivalent in both sub populations of CD11b^+^GR-1^hi^ myeloid cells. Similar to the published descriptions of MDSC subtypes isolated from spleens of tumor-bearing mice [17], [18], both granulocytic (Ly6G^+^Ly6C^dim^) and monocytic (Ly6G^−^Ly6C^bright^) BM-derived IMCs expressed Ly-6C (Fig. 2).

### Suppressive capacity of naïve BM-derived CD11b^+^GR-1^+^ IMCs is comparable with MDSCs from tumor-bearing mice

**Figure 3:**
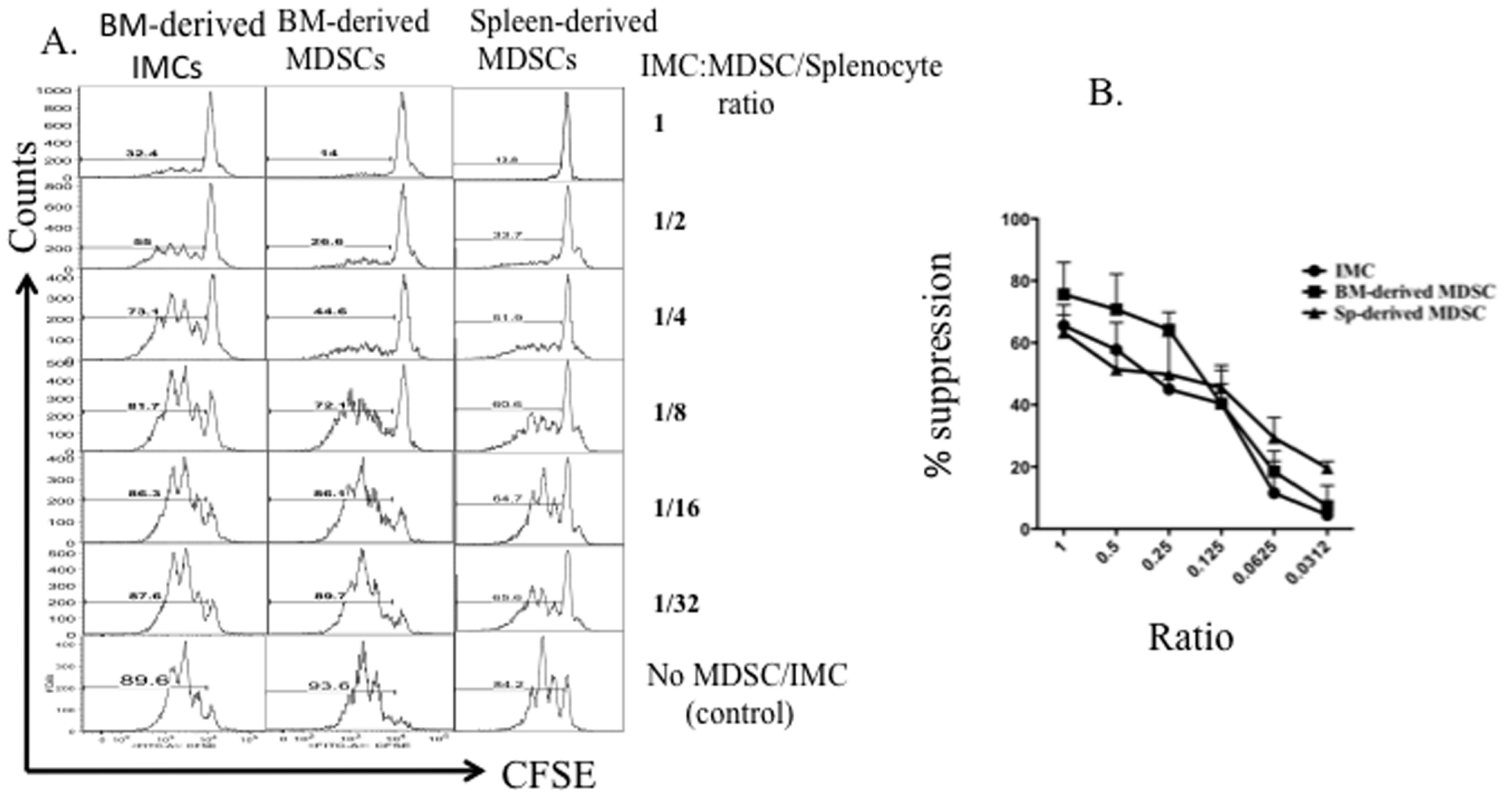
Comparison of suppressive activity of MDSCs with BM-derived IMCs. Bone marrow was harvested from 4T1 tumor-bearing BALB/c mice with 34-day primary tumors (10–15 mm in diameter) and from tumor-free BALB/c mice. Cells were stained using fluorochrome-conjugated antibodies against CD11b, GR-1, and lineage markers as described in Materials and Methods. Splenocytes from BALB/c mice were stained with 1 µm CFSE and 1×10^6^ cells per well of a 24-well dish, were stimulated with anti-CD3/anti-CD28 Dynabeads and IL-2, and co-cultured for 5 days with sorted MDSCs and IMCs from bone marrow of tumor or non-tumor bearing mice respectively, then analyzed by flow cytometry. A) The CFSE profile of CD8^+^ T-cell cultured with decreasing numbers of MDSCs or IMCs. B) Percent inhibition of proliferation at different T-cell: MDSC or IMC ratios. Representative data from three individual experiments is shown.

A variety of studies have reported that MDSCs in tumor-bearing animals have immune-suppressive effects on T-cell proliferation [9], [19], [20]. To compare suppressive activity of CD11b^+^GR-1^+^ IMCs isolated from the BM of non-tumor bearing mice with BM and spleen-derived MDSCs from tumor-bearing animals, we sorted myeloid progenitors from tumor-bearing and non-tumor-bearing mice and determined their suppressive activity by titrating ratios of myeloid cells: T-cells and measuring T-cell proliferation *in vitro*. We harvested BM and spleen from non-tumor-bearing BALB/c mice and 4T1-tumor-bearing mice and sorted CD11b^+^GR-1^+^ myeloid progenitors. BM-derived IMCs from non- tumor bearing mice and MDSCs from tumor-bearing mice were co-cultured with CFSE-labeled splenocytes in the presence of anti CD3/CD28 Dynabeads, and T-cell proliferation analyzed by flow cytometry after 5 days of culture. The suppressive activity of BM-derived MDSCs from tumor-bearing mice and BM-derived IMCs from non-tumor bearing mice was similar based upon inspection of the CFSE staining histogram profiles of CD8^+^ T-cells (Fig. 3A). Based upon the titration of myeloid cells: T-cells in culture, CD11b^+^GR-1^+^ IMCs from non-tumor-bearing mice and CD11b^+^GR-1^+^ MDSCs from tumor-bearing mice had nearly equivalent potency as assessed by the myeloid: T-cell ratio sufficient to achieve half-maximal suppression of T-cell proliferation (Fig. 3B).

### CD11b^+^GR-1^+^ IMCs induce T-cell apoptosis

**Figure 4:**
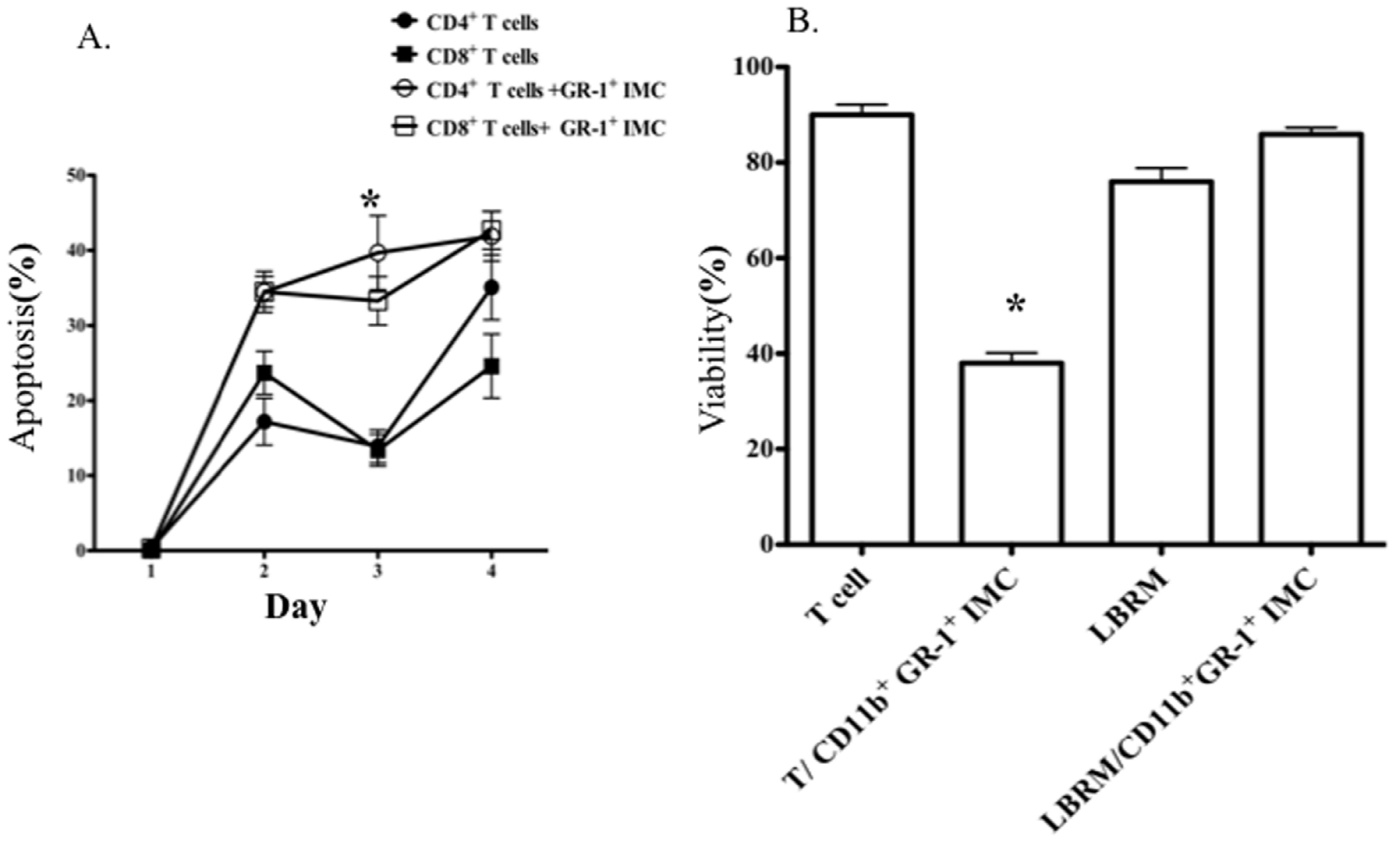
Co culture of Dynabead activated T-cells with CD11b^+^ GR-1^+^ IMC induced T-cell apoptosis. Left panel: frequencies of 7-AAD (+)/Annexin V (+) CD4^+^ and CD8^+^ T-cells cultured in the absence (filled symbols) or presence of CD11b^+^GR-1^+^ IMCs (empty symbols), as determined by flow cytometry. Right panel: Viability of T-cells and the T-cell lymphoblastic cell line, LBRM, cultured in the presence and absence of CD11b^+^ sorted IMCs Trypan blue staining was performed after 4 days of culture (*p* = 0.0088). Data represent of mean values (± SD) of three experiments.

Several studies have demonstrated that macrophages isolated from tumor-bearing mice may induce apoptosis of T-cells [21]–[23]. The ability of CD11b^+^GR-1^+^ IMCs from non-tumor-bearing B6 mice to induce T-cell apoptosis was assessed by measuring Annexin V and 7-AAD staining in Dynabead-stimulated T-cells cultured for 1, 2, 3 and 4 days with purified IMCs. The majority of T-cells (76%±2.1 of CD8^+^ and 64%±4.94 of CD4^+^ T-cells) had undergone apoptosis after 3 days co-culture with CD11b^+^GR-1^+^ IMCs compared to 48%±5% of CD8^+^ and 29%±2% of CD4^+^ T-cells cultured alone with anti CD3/CD28 beads (*p*<0.05) (Fig. 4A)(Fig. S1 D& E). There was no correlation between T-cell division and expression of Fas, FasL, CD27 or 4IBBL on T-cells (data not shown), suggesting that T-cell apoptosis in co-cultures with CD11b^+^GR-1^+^ IMCs did not result from activation-induced cell death. To test whether CD11b^+^GR-1^+^ IMCs had a general cytotoxic effect on lymphoid cells, we co-cultured a T-cell lymphoblastic lymphoma cell line LBRM with sorted IMCs and measured viability and proliferation of LBRM detected using Trypan Blue staining. IMCs did not inhibit proliferation or induce apoptosis of LBRM, indicating that the ability of IMCs to inhibit T-cell proliferation and induce apoptosis is not a generalized effect on dividing cells (Fig. 4B).

### Neutralizing antibody to IL-4 and IFN-γ KO IMCs partially abrogate IMC-suppression

**Figure 5:**
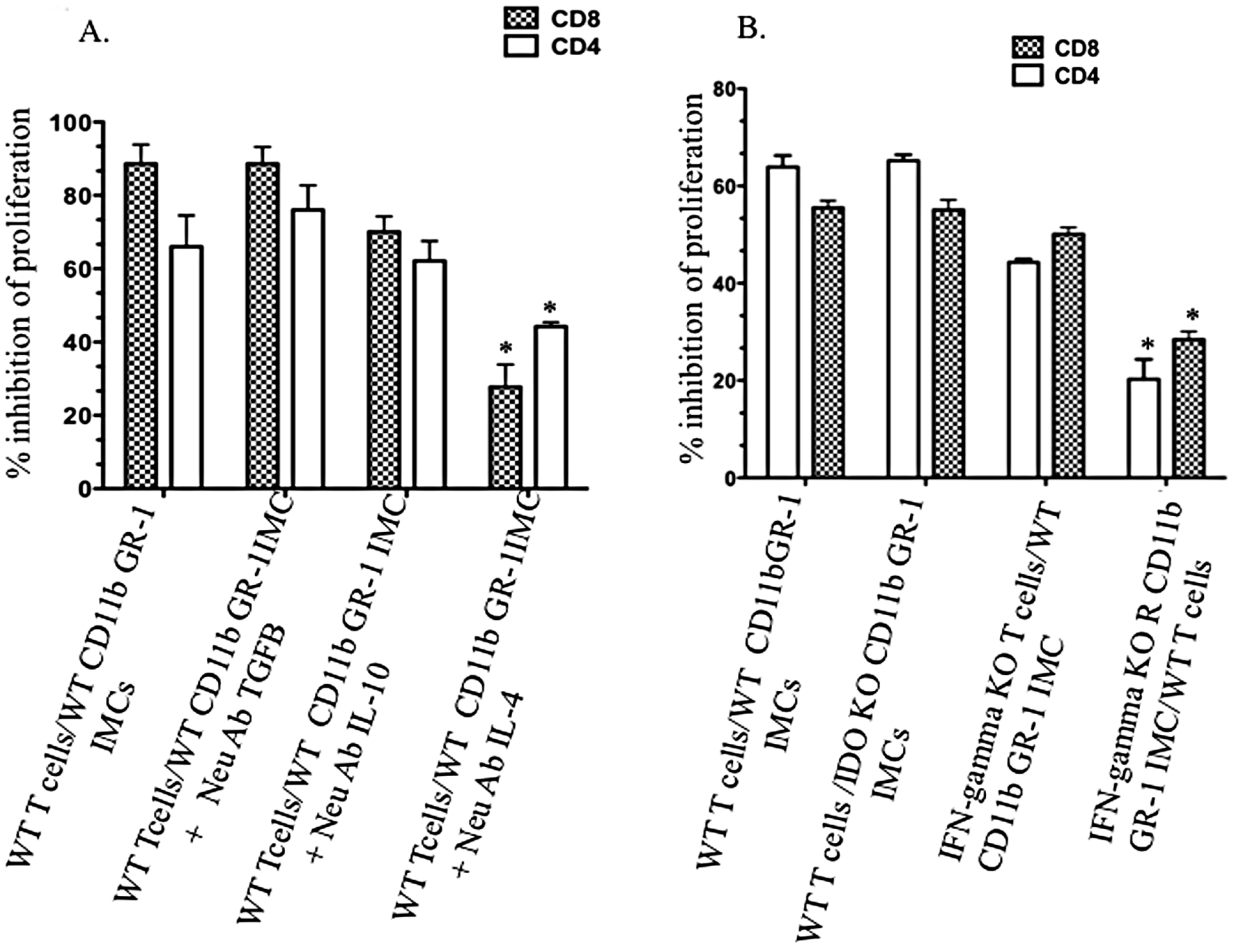
Partially abrogation of IMC-suppression by IL-4 neutralizing antibody and IFN-γ KO IMCs. A) Inhibition of Dynabead-induced proliferation of T-cells in co-culture with CD11b^+^GR-1^+^ IMCs was measured in the presence of neutralizing antibodies to TGFβ *(p>0.05)*, IL-4 (*p* = 0.0103), and IL-10 (*p>0.05*). B) Inhibition of proliferation of T-cells in co-culture with CD11b^+^GR-1^+^ IMCs isolated from wild-type mice (controls) or knock out mice for IDO *(p>0.05)*, and IFN-γ receptor (*p* = 0.0358). Additional combination used wild-type IMCs co cultured with IFN-γ KO T-cells. Data represent the mean and SD of three experiments.

Several publications, using a variety of murine tumor models, have identified IL-4 and TGFβ-dependent suppressive effects of tumor associated MDSCs [24], [25]. To assess the possible roles of these cytokines in the suppressive activity of IMCs in the bone marrow of non-tumor-bearing mice, we added neutralizing antibodies to IL-4, IL-10 and TGFβ1 to cultures of mitogen activated T-cells co-cultured with CD11b^+^GR-1^+^ IMC. A partial restoration of T-cell proliferation was seen following the addition of anti-IL-4 antibody while neutralizing antibodies to TGFβ1 and IL-10 did not restore T-cell proliferation (Fig. 5A). Next we studied the roles for IDO, IFN-γ and IL-12 in the suppressive activity of IMCs, since these molecules are also known to be involved in immunosuppressive signaling [26]–[28]. IMCs from IL-12 knock-out mice (data not shown), and IDO knock out mice had inhibitory capacity similar to wild type IMCs, and T-cells from IFN-γ KO mice did not show reduced proliferation in the presence of wild-type IMCs. In contrast, IMCs from IFN-γ receptor KO mice were substantially less potent in inhibiting T-cell proliferation (Fig. 5B). We did not detect significant expression of Foxp3 on CD4^+^/CD25^+^ T-cells after 4–5 days co-culture with IMCs; suggesting IMC-mediated suppression is not through the induction or *de novo* generation of T-Regulatory cells (data not shown).

### IFN-γ production by T-cells licenses NO production by CD11b^+^ GR1^+^ IMCs

**Figure 6:**
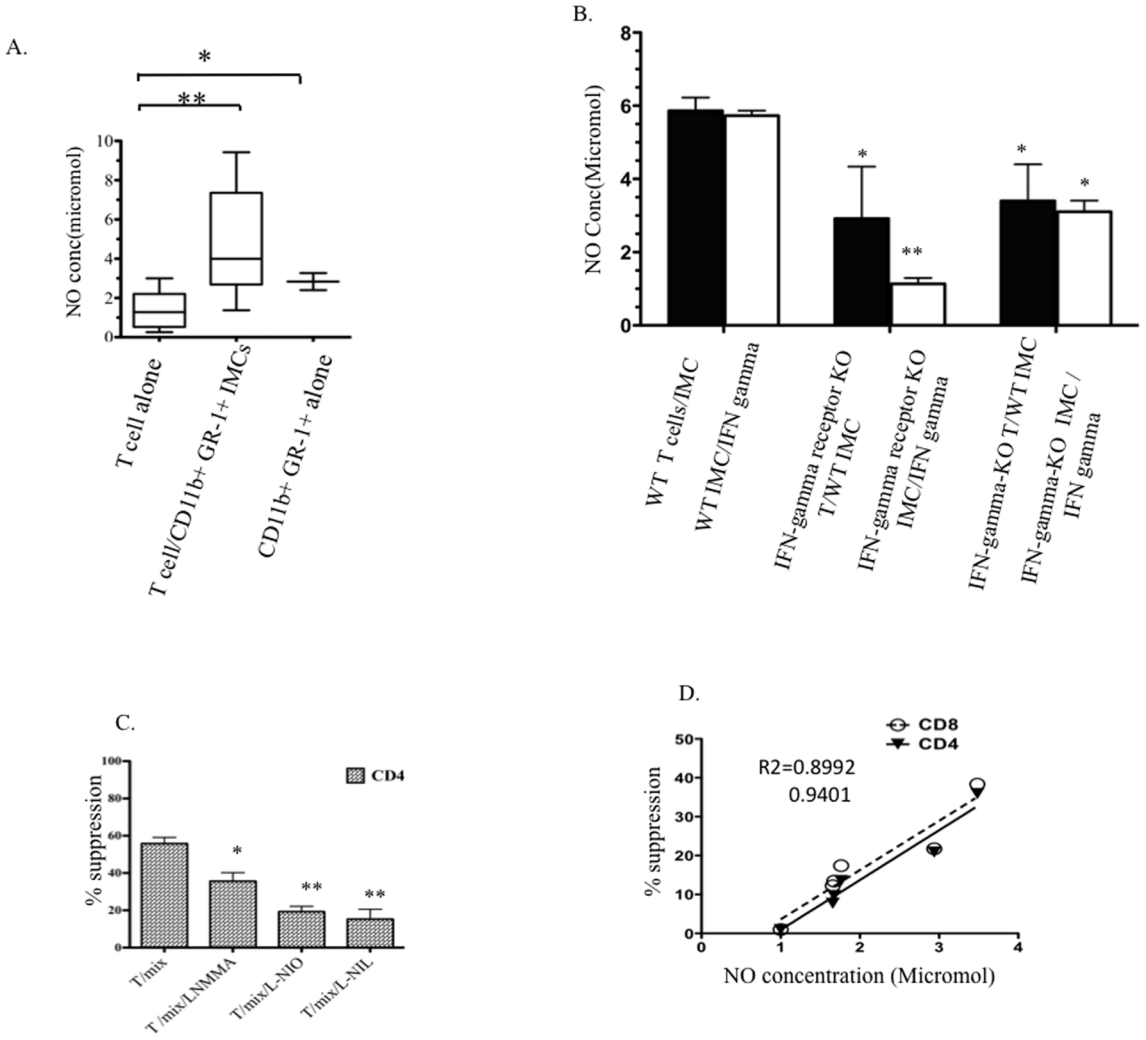
T-cell inhibition is mediated by an IFN-γ/NO pathway. Supernatants were collected after 4–5 days of co culturing Dynabead-activated T-cells with CD11b^+^GR-1^+^ immature myeloid cells sorted from BM. A) NO concentration in the supernatants of wild-type T-cells with and without wild-type IMCs (*p* = 0.0033). B) NO concentration in the supernatants of combinations of wild- type T-cells with IMCs, wild type T-cells with IFN-γ receptor KO IMCs, and IFN-γ KO T-cells with wild type IMCs (black bars from left to right). White bars show NO concentrations in supernatant of IFN-γ (50 ng/ml) treated IMCs cultures FACS-purified from wild type, IFN-γ receptor KO, and IFN-γ KO BM. Data represent mean and SD of four experiments. C) Inhibition of Dynabead-induced proliferation of CD4^+^ T-cells with mix of both subunits CD11b^+^GR-1^hi^ and GR-1^low^ in the presence and absence of NO inhibitors *p* = 0.0419. D) Correlation of NO production and inhibition of proliferation in co-cultures containing different ratios of CD11b^+^GR-1^+^ IMCs: T-cells. Solid and dashed lines represent best-fit correlation of NO concentration with inhibition of proliferation of CD4^+^ and CD8^+^ T-cells. (* Indicates *p<0.05*, ** indicates *p<0.001*).

As nitric oxide (NO) is considered to be a key component in T-cell suppression mediated by MDSCs [29], [30], we measured the role of NO in co-cultures of T-cells and IMCs to determine whether NO serves as a short-range soluble mediator that could inhibit T-cell proliferation. Extracellular NO was significantly higher in cultures of T-cells co-cultured with CD11b^+^GR1^+^ IMCs compared with media from cultures of Dynabead activated T-cells alone (Fig. 6A). To confirm the effect of IMC on NO produced in co-cultures of T-cells and IMC, we measured extracellular NO concentration in media of co-cultures of T-cells with CD11b^+^GR1^+^ IMCs in which the numbers of IMCs were progressively decreased (Fig. S4) and used flow cytometry to measure intracellular NO in IMCs and T-cells from co-cultures (Fig. S5). Our results showed that co-culture of CD11b^+^GR-1^+^ with T-cells lead to enhanced NO production in the IMC population. Consistent with the previously described role for IFN-γ signaling in the immuno-suppressive activity of MDSCs, extracellular NO was significantly lower in supernatants of co-cultures of wild-type T-cells and CD11b^+^ GR1^+^ IMCs isolated from IFN-γ receptor KO mice compared with supernatants of IFN-γ KO T-cells cultured with wild type IMCs (Fig. 6B, filled bars). To better understand the relationship between IFN-γ signaling, NO and suppression of T-cell proliferation, we measured NO production after adding IFN-γ (50 ng/ml) to the same T-cell/IMC co-cultures described above (white bar graphs left to right). Our results show significantly less NO production by IFN-γ receptor KO CD11b^+^ GR1^+^ IMCs than wild type IMCs, demonstrating a role for IFN-γ signaling receptors on IMCs in NO production. Adding iNOS inhibitors (L-NMMA, L-NIO, and L-NIL) to co-cultures of T-cells with IMCs restored T-cell proliferation (Fig. 6C). Measurement of extracellular NO in the culture media from multiple experiments showed a high correlation between NO and the degree to which T- cell proliferation was inhibited (Fig. 6D).

### Viability and cell-to-cell contact are required for BM-derived CD11b^+^GR1^+^ IMC mediated suppression

**Figure 7:**
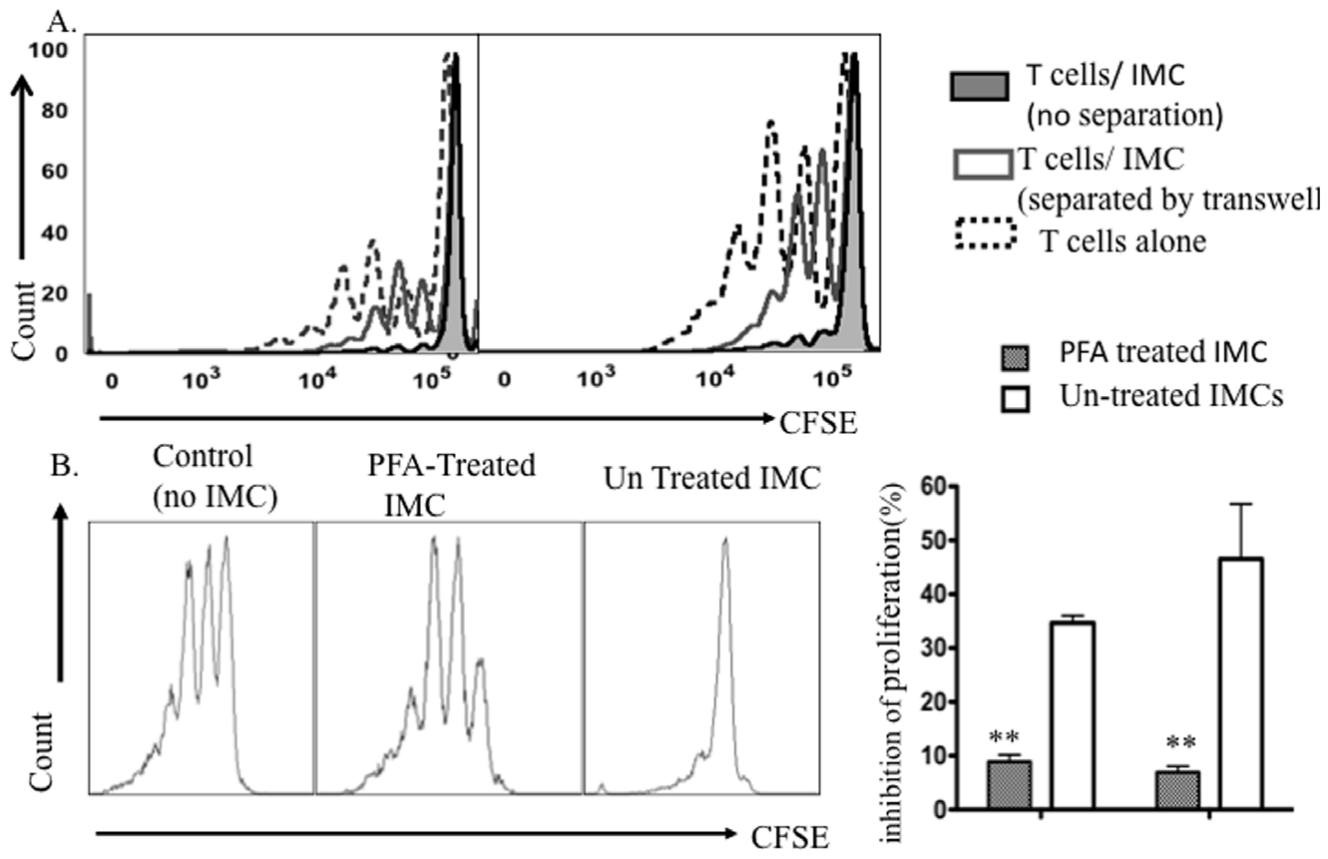
Viability of IMCs & cell-cell contact is required for suppression of T-cell proliferation. A) CFSE fluorescence histograms of purified T-cells activated with Dynabead cultured alone or co-cultured with sorted CD11b^+^ GR-1^+^ IMCs in regular and Transwell plates at a 1∶1 ratio for 4 days. Data are representative of three experiments. B) CFSE fluorescence histograms of viable CD4^+^ T-cells after 4 days co-culture with PFA- treated CD11b^+^GR-1^+^ IMC (middle panel); untreated IMCs (right panel) versus T-cells alone (left panel). C) Mean values ±SD for inhibition of proliferation of CD4^+^ and CD8^+^ T-cells after culture with PFA- treated IMCs compared with untreated culture IMCs (*p<0.001*). Data from three individual experiments.

Given the close proximity of IMCs with T-cells in the bone marrow microenvironment, we next asked whether cell-cell contact between T-cells and IMC was required for suppressing T-cell proliferation. Physical separation of T-cells and IMCs in a Transwell culture nearly completely abrogated the suppressive activity of bone marrow IMCs. The percentage of divided T-cells after activation by Dynabeads increased by 5-fold compared with T-cells and IMCs co-cultured in the same culture chamber, indicating that direct T-cell-to cell contact or the production of short-range soluble mediators are required for the suppressive effect of IMCs (Fig. 7A). We tested whether viability of IMCs was required for their observed suppressive activity by fixing IMCs with PFA. Fixation of IMCs significantly abrogated their suppressive activity on mitogen activated T-cells (Fig. 7B). Taken together, these data indicate that suppression of T-cell proliferation requires contact with live IMCs, likely due to signaling through cell surface receptors or short-range soluble mediators.

## Discussion

This work was undertaken to characterize the ability of myeloid precursors from the BM of non-tumor-bearing mice to inhibit T-cell proliferation. In contrast to previous studies that suggested BM-derived CD11b^+^GR-1^+^ IMCs from tumor-free mice lack immunosuppressive activity [8], this is the first study to definitively document that CD11b^+^GR-1^+^ IMCs isolated from the BM of non tumor-bearing mice have comparable ability to suppress T-cell proliferation as MDSCs from tumor-bearing mice. While previous reports characterized the suppressive effect of CD11b^+^GR-1^+^ from cytokine-activated bone marrow and spleen [26], [31], [32], and the immunosuppressive activity of CD11b^+^ Ly6C^+^ Ly6G^−^ mononuclear cells in the blood of non-tumor-bearing mice [33], and multiple hematopoietic tissues (BM, blood, spleen, and peripheral lymph nodes) of healthy aged mice [34], we studied IMCs purified from non-stimulated bone marrow by FACS. It is likely that the administration of cytokines changes the immuno-modulatory properties of myeloid progenitors [35], and that granulocytic cells isolated from the blood have different immuno-modulatory properties than immature myeloid cells in the bone marrow. Of note, the BM-derived CD11b^+^GR-1^+^ IMCs from non-tumor-bearing mice expressed a different pattern of cell surface markers than splenocyte derived MDSCs from tumor-bearing mice (Fig. S3). Specifically, IMC lack expression of CD124 and PDL-1, two markers associated with immuno-suppression [18], [36], [37]. These results indicate that tumor-associated signals can lead to specific patterns of differentiation of myeloid progenitors, compared with IMCs isolated directly from bone marrow. Our findings indicate that the suppression activity of CD11b^+^GR-1^+^ IMCs is mediated *in vitro* (in part) through local production of NO that suppresses T-cell proliferation. Of note, apoptosis of T-cells co-cultured with IMCs was induced, while LBRM, a T-cell lymphoma cell line, co-cultured with IMC did not undergo apoptosis. The different susceptibility of normal T-cells versus malignant T-cells to IMC-induced apoptosis may reflect intrinsic anti-apoptotic mechanisms activated in the malignant T-cell population. Previous studies showed T-cells could be a source of NO production [30]. Our findings showed that co-culture of immature myeloid cells with T-cells leads to enhanced NO production in the IMC population. Abrogation of the suppressive effect of IMCs on T-cell proliferation using Transwell cultures to separate T-cells from IMCs, or by the use of NO inhibitors, support a role for release of short-range soluble mediators (such as NO) by IMCs that inhibit T-cell proliferation. We found a critical role for IFN-γ as a licensing cytokine leading to iNOS activation and inhibition of T-cell proliferation. NO was lower in culture media using IFN-γ KO T-cells with cultured wild type IMCs or wild type T-cells cultured with IFN-γ receptor KO IMCs compared with cocultures of wild type T-cells and IMCs. Furthermore, T-cells from both IFN-γ KO and IFN-γ receptor KO mice produced lower amounts of NO compared with wild type T-cells, supporting a paracrine function of IFN-γ in NO production by T-cells. The downstream effects of IFN-γ signaling on NO production are also supported by the significant correlation between NO and the inhibition of T-cell proliferation. Our findings with IMCs from normal BM are consistent with published work on MDSCs from tumor-bearing animals that have established the role for IFN-γ in suppressing T-cell proliferation [26], [38]. In contrast to previous reports of a critical role for IDO or IL-12 pathways in the suppressive effect of MDSCs in tumor models [27], [39], we did not identify the requirement for these pathways in the suppressive effect of IMCs from non-tumor-bearing mice on T-cell proliferation. However, blocking the activity of IL-4 partially restored T-cell proliferation, suggesting that IL-4 signaling may suppress T-cell proliferation independent of the iNOS/NO pathway. Of note, there may be a role for other soluble mediators like prostaglandin E2 (PGE2), that regulate T-cell proliferation as physiological mediator of cell survival and differentiation via receptors are present on murine and human hematopoietic stem and progenitor cells [40] and on MDSCs [41]. Further experiments need to clarify possible roles for mediators like PGE2 in the immuno-suppressive activity of IMCs.

Taken together, these findings suggest that BM-derived IMCs may have an important physiological role in creating and maintaining an immunosuppressive state for T-cells in the bone marrow microenvironment. Our findings are consistent with reports indicating qualitative differences in the alloreactivity of T-cells isolated from BM versus splenic T-cells and their contribution to an immunosuppressive microenvironment [42], [43]. Further experiments might help to elucidate the immunoregulatory mechanisms of IMCs in maintaining an immuno-suppressive state in the BM microenvironment *in vivo*.

## Supporting Information

Figure S1 **IMCs inhibit activation of Dynabeads activated T-cells.** Freshly sorted naïve BM-derived CD11b^+^ GR-1^+^ cells and CFSE stained naïve-spleen derived T-cells were co cultured in presence of anti-CD3/CD28 beads. The activation status of cultured T-cells was determined based on CD25 staining after 5 days. A) Top panel shows the CFSE profile of cultured CD4^+^ T-cells in the presence and absence of CD11b^+^ GR-1^+^ IMCs; the lower panel shows corresponding data on CD8^+^ T-cells. B) The percentage of viabile T-cells cultured alone is compared to T-cells cultured with IMCs. C) The number of viable cells as a percentage compared to baseline values following 4 days culture of T-cells with or without CD11b^+^ GR-1^+^ IMCs. Viability of cultured cells was determined by Trypan blue staining. D) Left panel: CD4^+^ and CD8^+^ staining of T-cells. Middle panels: Annexin V and 7-AAD of CD4^+^ T-cells from T-cells cultured alone (top) and T-cells co-cultured with IMCs (bottom). Right panel: CFSE profile of viable 7-AAD (−), Annexin V (−) T CD4^+^ cells after 4 days of culture. E) Flow cytometry analysis of CD11b^+^ GR-1^+^ IMCs on day 4 co-cultures with T-cells. Left panel: scatter profile; Middle panel: CD11b^+^ IMCs from co-culture; Left panel: Annexin V and 7-AAD staining of CD11b^+^ IMCs following 4 days of culture.
(TIF)

Figure S2 **IMCs inhibit Ki-67 expression in T-cells were co-cultured with anti CD3/CD28 beads.** CFSE-labeled T-cells from wild type mouse spleen were co-cultured with FACS sorted BM-derived CD11b^+^GR-1^+^ IMCs at a ratio 1∶1. T-cells in the cultures were stimulated with anti-CD3/CD28 beads and IL-2 for 4 days. A) The CFSE profile of CD4^+^ T after intracellular Ki-67 staining comparing T- cells cultured alone with T-cells co-cultured with naïve BM-derived sorted CD11b^+^GR-1^+^ IMCs. B) The relative number of CFSE-divided and un-divided T-cells following stimulation with anti CD3/CD28 beads or after co-culture with CD11b^+^ GR-1^+^ IMCs and anti CD3/CD28 beads (p<0.05).
(TIF)

Figure S3 **Immunophenotype of 4T1 Bone marrow-derived MDSCs.** A) Flow cytometry analysis of cell surface marker expression on 7-AAD (−) BM-derived CD11b^+^GR-1^hi^ and CD11b^+^GR-1^low/int^ MDSC subsets from female BALB/c 28 days after 4T1 breast tumor inoculation was performed as described in Methods. B) Histograms represent expression of the indicated markers on viable CD11b^+^GR-1^+^MDSCs (open dashed histograms) compared with staining of gated MDSC population with an isotype control (filed gray histograms).
(TIF)

Figure S4 **NO concentration in media following co-culture of graded numbers of CD11b^+^ GR-1^+^ IMCs and T-cells.** Freshly naïve BM-derived sorted CD11b^+^ GR-1^+^ IMCs cells and T-cells co-cultured for 4 days. Supernatants were assayed for NO content as described in Methods.
(TIF)

Figure S5 **BM-derived IMCs inhibit intracellular NO production by activated T-cells.** Splenocyted-derived T-cells were activated with anti CD3/CD28 beads and co-cultured in presence and absence of sorted purified BM-derived CD11b^+^ GR-1^+^ cells. After 4 days of culture cells were stained for DAF and incubated for 45 minutes at37°C. NO production within viable (7-AAD negative) gated cells was analyzed as positive DAF staining versus control group without DAF stain. A) Flow cytometry histogram of intracellular NO level in CD11b^+^GR-1^+^ IMCs, representative of three individual experiments. B) Graphs showing mean fluorescence index (MFI) of DAF staining for T- cells co-cultured with CD11b^+^GR-1^+^ IMCs and CD11b^+^GR-1^+^ IMCs alone versus IMCs co-cultured with T-cells. Co-cultured cells not stained with DAF were used as a negative control.
(TIF)
