# Supplementary figures and images for: Properties of Immature Myeloid Progenitors with Nitric-Oxide-Dependent Immunosuppressive Activity Isolated from Bone Marrow of Tumor-Free Mice
Source: PLoS One. 2013 Jul 2;8(7):e64837. doi: 10.1371/journal.pone.0064837 (PMC3699563; doi:10.1371/journal.pone.0064837)

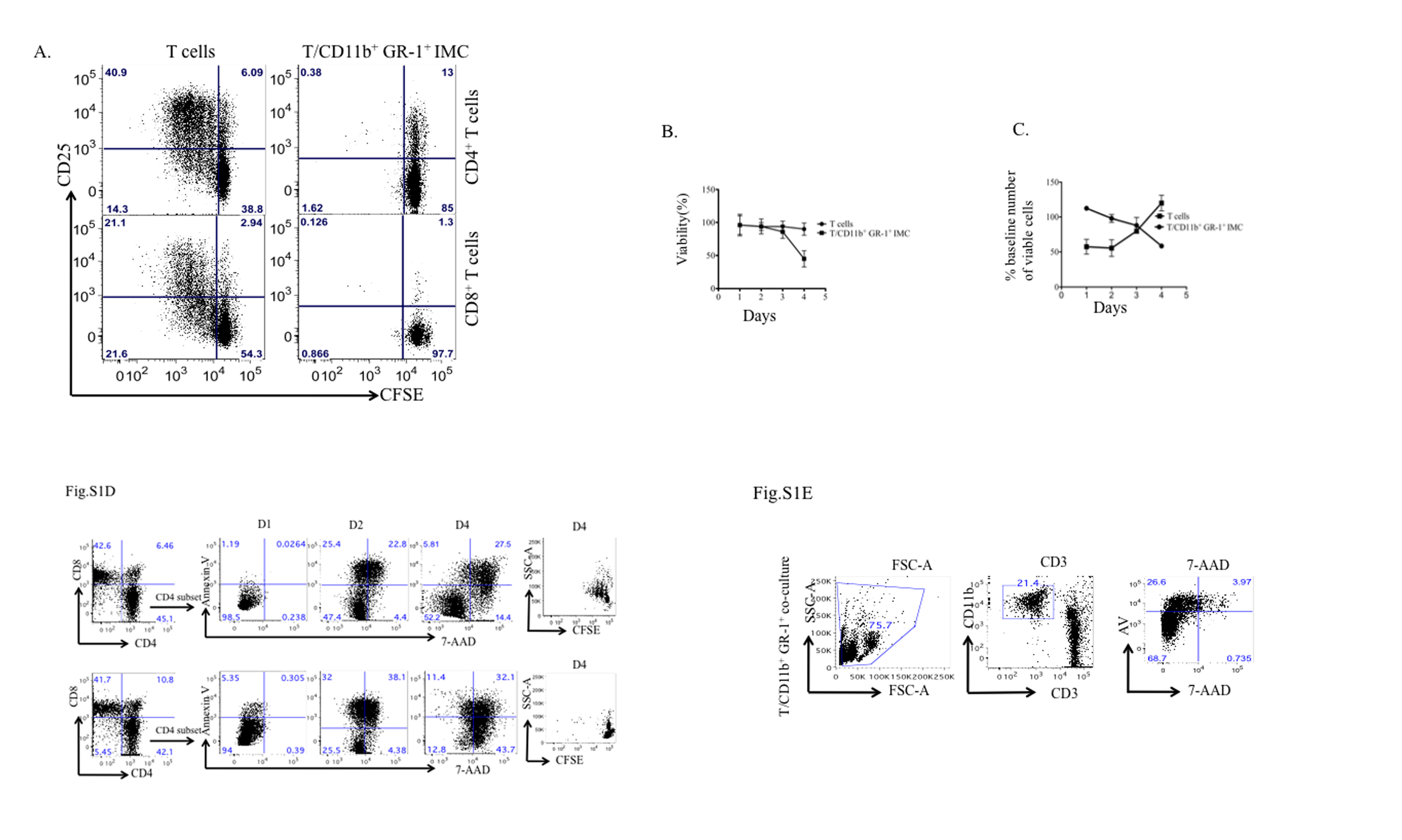

Supplement: Figure S1 — IMCs inhibit activation of Dynabeads activated T-cells. Freshly sorted naïve BM-derived CD11b+ GR-1+ cells and CFSE stained naïve-spleen derived T-cells were co cultured in presence of anti-CD3/CD28 beads. The activation status of cultured T-cells was determined based on CD25 staining after 5 days. A) Top panel shows the CFSE profile of cultured CD4+ T-cells in the presence and absence of CD11b+ GR-1+ IMCs; the lower panel shows corresponding data on CD8+ T-cells. B) The percentage of viabile T-cells cultured alone is compared to T-cells cultured with IMCs. C) The number of viable cells as a percentage compared to baseline values following 4 days culture of T-cells with or without CD11b+ GR-1+ IMCs. Viability of cultured cells was determined by Trypan blue staining. D) Left panel: CD4+ and CD8+ staining of T-cells. Middle panels: Annexin V and 7-AAD of CD4+ T-cells from T-cells cultured alone (top) and T-cells co-cultured with IMCs (bottom). Right panel: CFSE profile of viable 7-AAD (−), Annexin V (−) T CD4+ cells after 4 days of culture. E) Flow cytometry analysis of CD11b+ GR-1+ IMCs on day 4 co-cultures with T-cells. Left panel: scatter profile; Middle panel: CD11b+ IMCs from co-culture; Left panel: Annexin V and 7-AAD staining of CD11b+ IMCs following 4 days of culture. (TIF) [file pone.0064837.s001.tif]

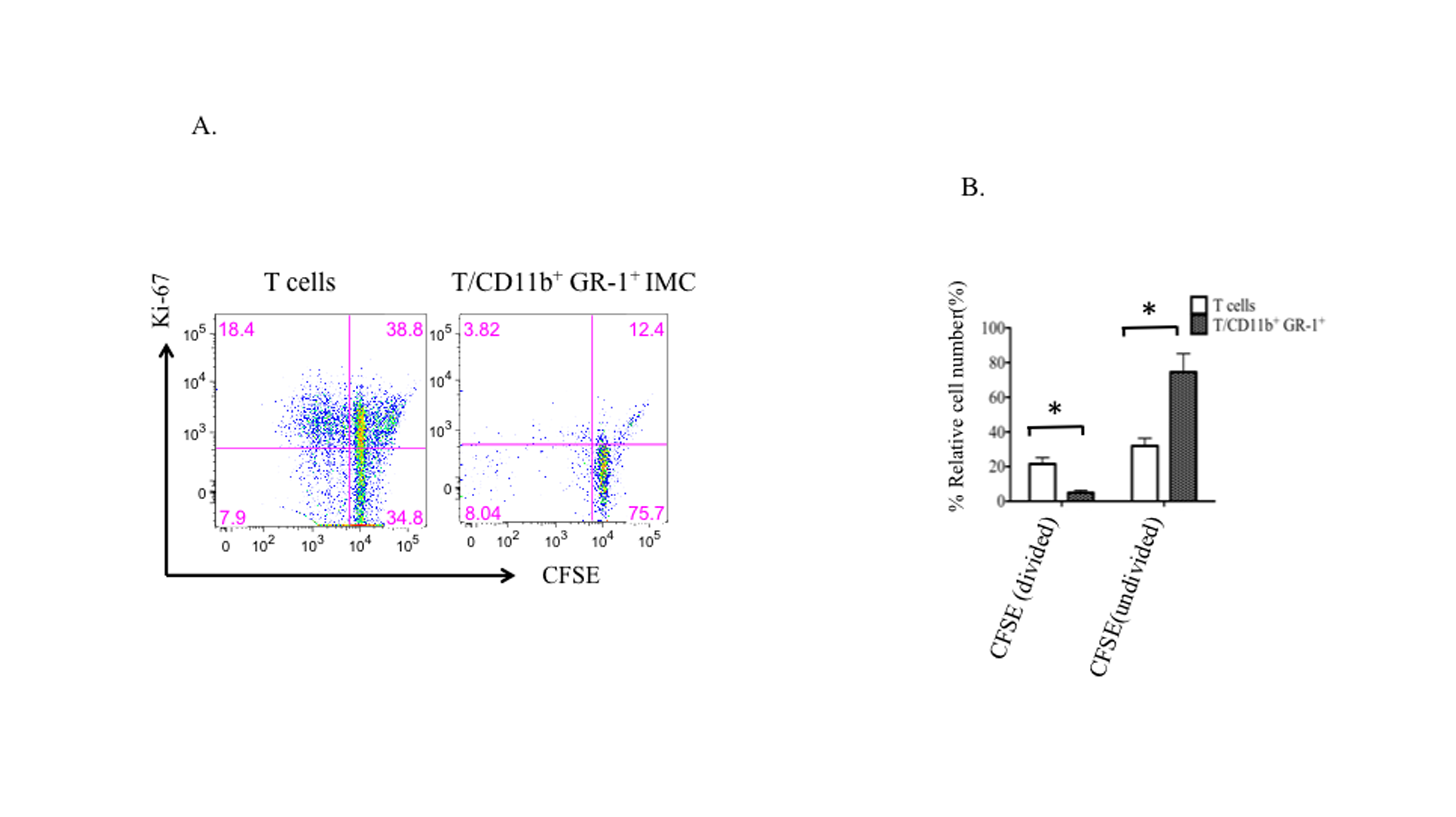

Supplement: Figure S2 — IMCs inhibit Ki-67 expression in T-cells were co-cultured with anti CD3/CD28 beads. CFSE-labeled T-cells from wild type mouse spleen were co-cultured with FACS sorted BM-derived CD11b+GR-1+ IMCs at a ratio 1∶1. T-cells in the cultures were stimulated with anti-CD3/CD28 beads and IL-2 for 4 days. A) The CFSE profile of CD4+ T after intracellular Ki-67 staining comparing T- cells cultured alone with T-cells co-cultured with naïve BM-derived sorted CD11b+GR-1+ IMCs. B) The relative number of CFSE-divided and un-divided T-cells following stimulation with anti CD3/CD28 beads or after co-culture with CD11b+ GR-1+ IMCs and anti CD3/CD28 beads (p<0.05). (TIF) [file pone.0064837.s002.tif]

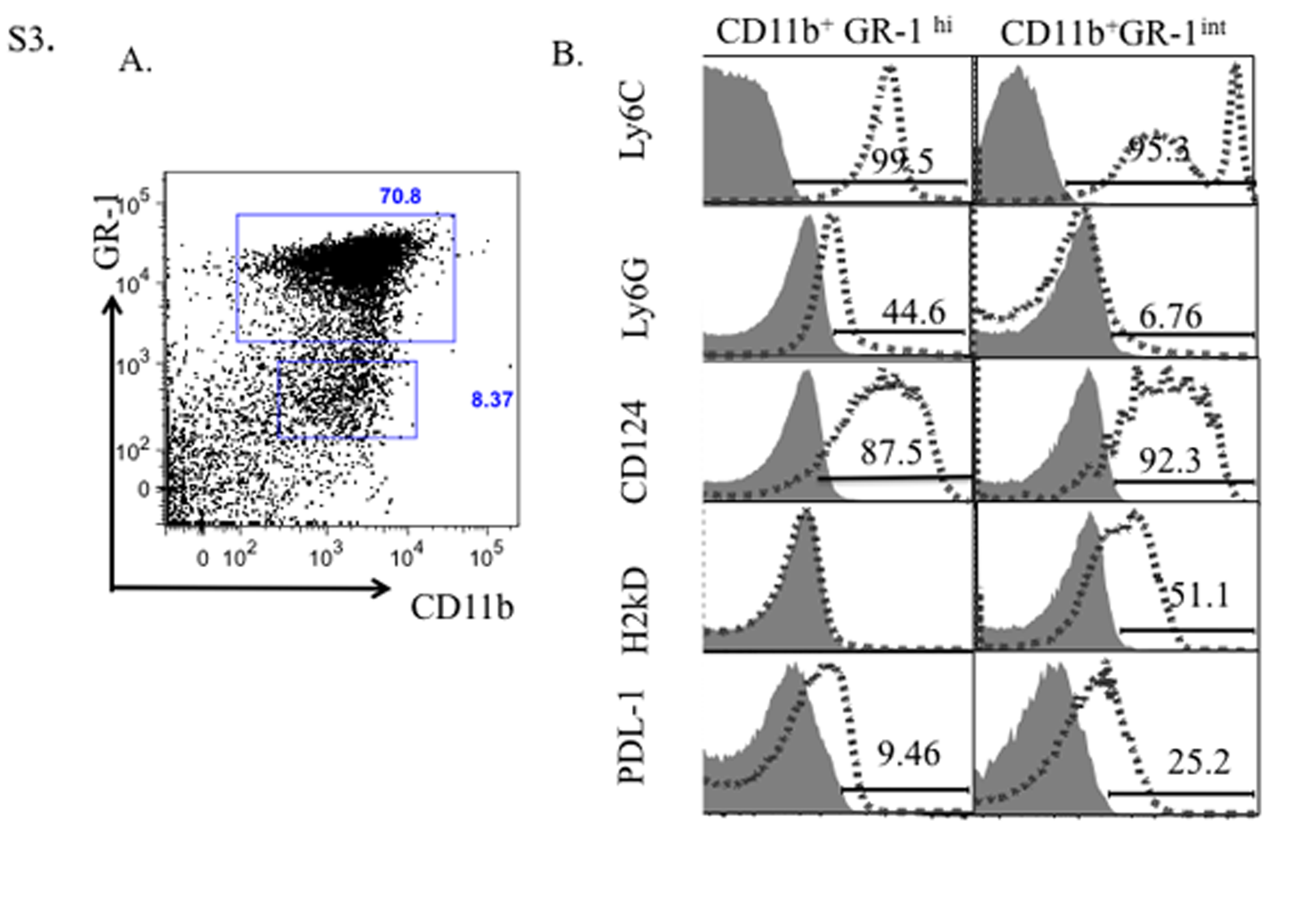

Supplement: Figure S3 — Immunophenotype of 4T1 Bone marrow-derived MDSCs. A) Flow cytometry analysis of cell surface marker expression on 7-AAD (−) BM-derived CD11b+GR-1hi and CD11b+GR-1low/int MDSC subsets from female BALB/c 28 days after 4T1 breast tumor inoculation was performed as described in Methods. B) Histograms represent expression of the indicated markers on viable CD11b+GR-1+MDSCs (open dashed histograms) compared with staining of gated MDSC population with an isotype control (filed gray histograms). (TIF) [file pone.0064837.s003.tif]

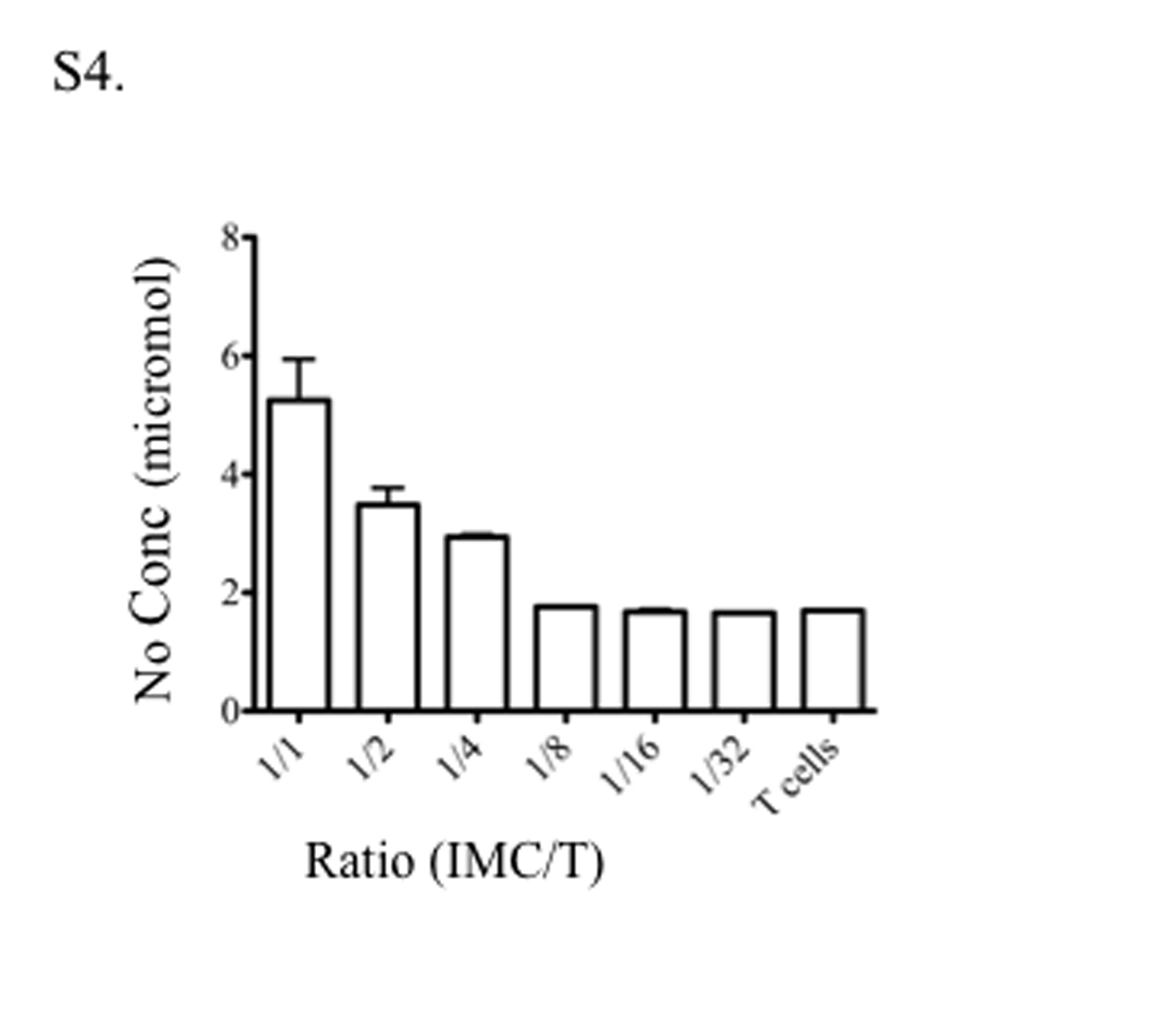

Supplement: Figure S4 — NO concentration in media following co-culture of graded numbers of CD11b+ GR-1+ IMCs and T-cells. Freshly naïve BM-derived sorted CD11b+ GR-1+ IMCs cells and T-cells co-cultured for 4 days. Supernatants were assayed for NO content as described in Methods. (TIF) [file pone.0064837.s004.tif]

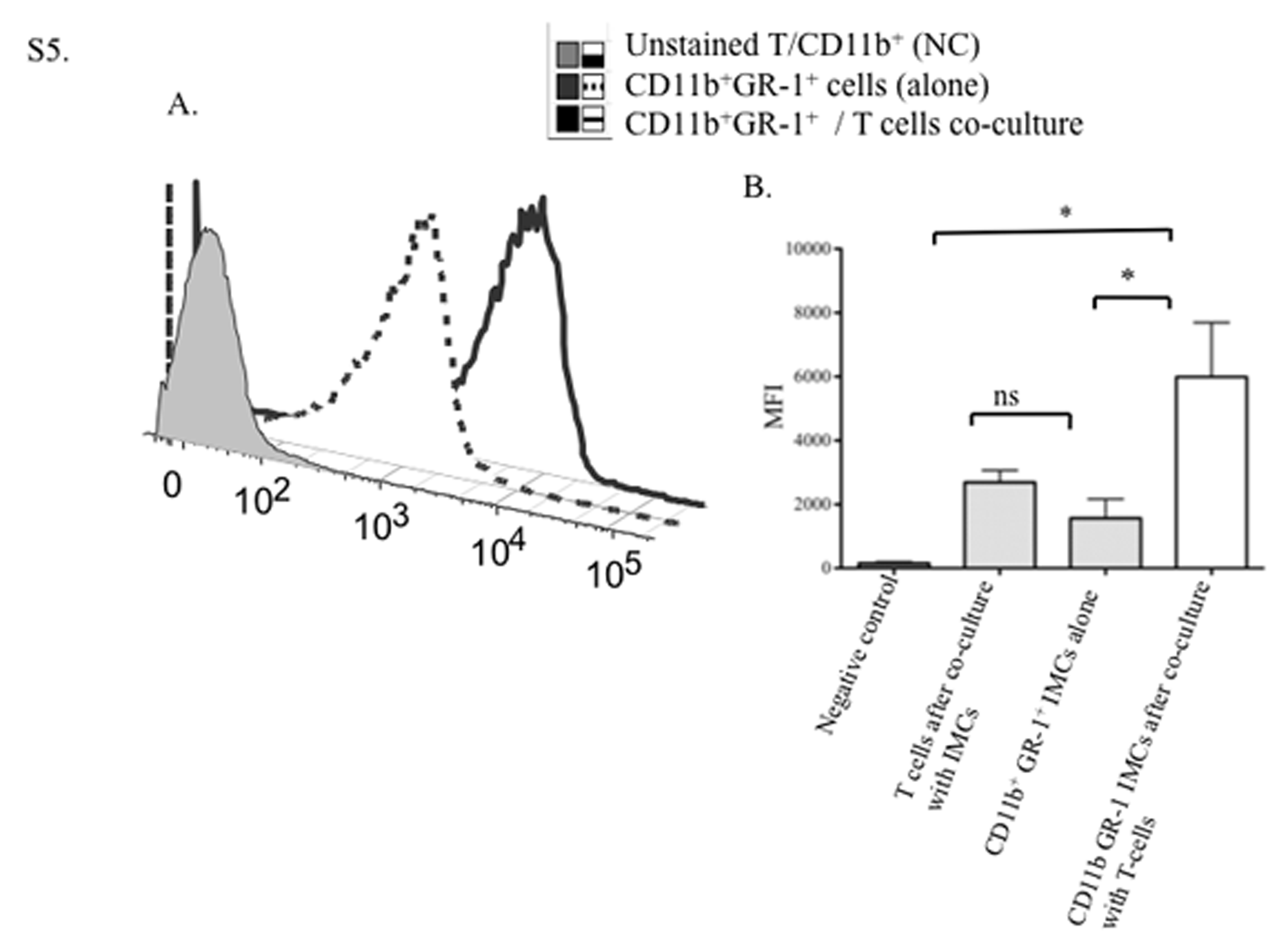

Supplement: Figure S5 — BM-derived IMCs inhibit intracellular NO production by activated T-cells. Splenocyted-derived T-cells were activated with anti CD3/CD28 beads and co-cultured in presence and absence of sorted purified BM-derived CD11b+ GR-1+ cells. After 4 days of culture cells were stained for DAF and incubated for 45 minutes at37°C. NO production within viable (7-AAD negative) gated cells was analyzed as positive DAF staining versus control group without DAF stain. A) Flow cytometry histogram of intracellular NO level in CD11b+GR-1+ IMCs, representative of three individual experiments. B) Graphs showing mean fluorescence index (MFI) of DAF staining for T- cells co-cultured with CD11b+GR-1+ IMCs and CD11b+GR-1+ IMCs alone versus IMCs co-cultured with T-cells. Co-cultured cells not stained with DAF were used as a negative control. (TIF) [file pone.0064837.s005.tif]
